# Rab8a and Rab8b are essential for several apical transport pathways but insufficient for ciliogenesis

**DOI:** 10.1242/jcs.136903

**Published:** 2014-01-15

**Authors:** Takashi Sato, Tomohiko Iwano, Masataka Kunii, Shinji Matsuda, Rumiko Mizuguchi, Yongwook Jung, Haruo Hagiwara, Yoshihiro Yoshihara, Michisuke Yuzaki, Reiko Harada, Akihiro Harada

**Affiliations:** 1 Institute for Molecular and Cellular Regulation, Gunma University, Maebashi, Gunma 371-8512, Japan; 2 Department of Cell Biology, Graduate School of Medicine, Osaka University, Suita, Osaka 565-0871, Japan; 3 Department of Physiology, School of Medicine, Keio University, Shinanomachi, Shinjuku-ku, Tokyo 160-8582, Japan; 4 Laboratory for Neurobiology of Synapse, RIKEN Brain Science Institute, Saitama 351-0198, Japan; 5 Department of Anatomy, Dongguk University, Suk-Jang Dong, Kyongju 780-714, Korea; 6 Department of Anatomy, Teikyo University School of Medicine, 2-11-1 Kaga, Itabashi-ku, Tokyo 173-8605, Japan; 7 Department of Judo Therapy, Takarazuka University of Medical and Health Care, Takarazuka, Hyogo 666-0162, Japan

**Keywords:** Rab8b, Rab8a, Knockout mouse, Cell polarity, Apical membrane, Cilia

## Abstract

The small GTP-binding protein Rab8 is known to play an essential role in intracellular transport and cilia formation. We have previously demonstrated that Rab8a is required for localising apical markers in various organisms. Rab8a has a closely related isoform, Rab8b. To determine whether Rab8b can compensate for Rab8a, we generated *Rab8b*-knockout mice. Although the *Rab8b*-knockout mice did not display an overt phenotype, *Rab8a* and *Rab8b* double-knockout mice exhibited mislocalisation of apical markers and died earlier than *Rab8a*-knockout mice. The apical markers accumulated in three intracellular patterns in the double-knockout mice. However, the localisation of basolateral and/or dendritic markers of the double-knockout mice seemed normal. The morphology and the length of various primary and/or motile cilia, and the frequency of ciliated cells appeared to be identical in control and double-knockout mice. However, an additional knockdown of Rab10 in double-knockout cells greatly reduced the percentage of ciliated cells. Our results highlight the compensatory effect of Rab8a and Rab8b in apical transport, and the complexity of the apical transport process. In addition, neither Rab8a nor Rab8b are required for basolateral and/or dendritic transport. However, simultaneous loss of Rab8a and Rab8b has little effect on ciliogenesis, whereas additional loss of Rab10 greatly affects ciliogenesis.

## INTRODUCTION

The small GTP-binding protein Rab8 is reported to be involved in basolateral transport in epithelial cells (Huber et al., 1993a; Ang et al., 2003; Henry and Sheff, 2008) and in dendritic transport in neurons (Huber et al., 1993b). Rab8 is also reported to be involved in trafficking of neurotransmitter receptors in postsynaptic terminals (Gerges et al., 2004) and the formation of outer photoreceptor segments in the retina (Moritz et al., 2001; Bachmann-Gagescu et al., 2011). We have shown that Rab8a, an isoform of Rab8, is essential for localizing apical proteins in the small intestine by analysing *Rab8a*-knockout (AKO) mice (Sato et al., 2007). In addition, AKO mice display a phenotype that is strikingly similar to that shown in the human disease microvillus atrophy. One of the genes responsible for this hereditary disease is myosin Vb (Müller et al., 2008), which binds Rab8 (Roland et al., 2007). Thus, Rab8 is closely associated with the pathogenesis of this disease. Recently, the human disease Bardet-Biedl syndrome (BBS) was determined to result from mutations of a group of genes within the BBSome complex (a complex of seven BBS proteins and BBIP10), which is associated with the formation of cilia (Nachury et al., 2007). Interestingly, Rabin8, a guanine nucleotide exchange protein that activates Rab8, binds this complex for ciliogenesis (Nachury et al., 2007; Westlake et al., 2011). Using different experimental systems, other groups independently reported that Rab8 is involved in the elongation of ciliary membranes (Yoshimura et al., 2007; Omori et al., 2008). Thus, Rab8 is currently regarded as being involved in the formation of cilia and the pathogenesis of BBS.

However, it has been difficult to determine the function of Rab8 for several reasons. Rab8 consists of the two closely related proteins Rab8a and Rab8b, which are encoded by different genes (Armstrong et al., 1996). Both proteins are highly similar in their sequence and are expressed at similar levels. Therefore, knocking out the *Rab8a* gene is insufficient to uncover the role of Rab8 because Rab8b might compensate for Rab8a. In addition, regarding Rab8, an apparent contradiction was observed in previous findings that describe its role in basolateral transport, and our findings that indicate it has a role in apical transport. Thus, to determine the function of Rab8, an examination of the function of Rab8b is crucial. In addition, Rab8 has been proposed as being essential for ciliogenesis and for the pathogenesis of ciliopathies, including BBS. Therefore, determining the functional role of Rab8 has both scientific and clinical importance. To determine its role in polarised transport in epithelial cells, neurons and in ciliogenesis, we generated *Rab8b* knockout (BKO) mice and *Rab8a* and *Rab8b* double-knockout (DKO) mice.

## RESULTS

### Rab8b knockout mice display no overt phenotype

**Fig. 1.**
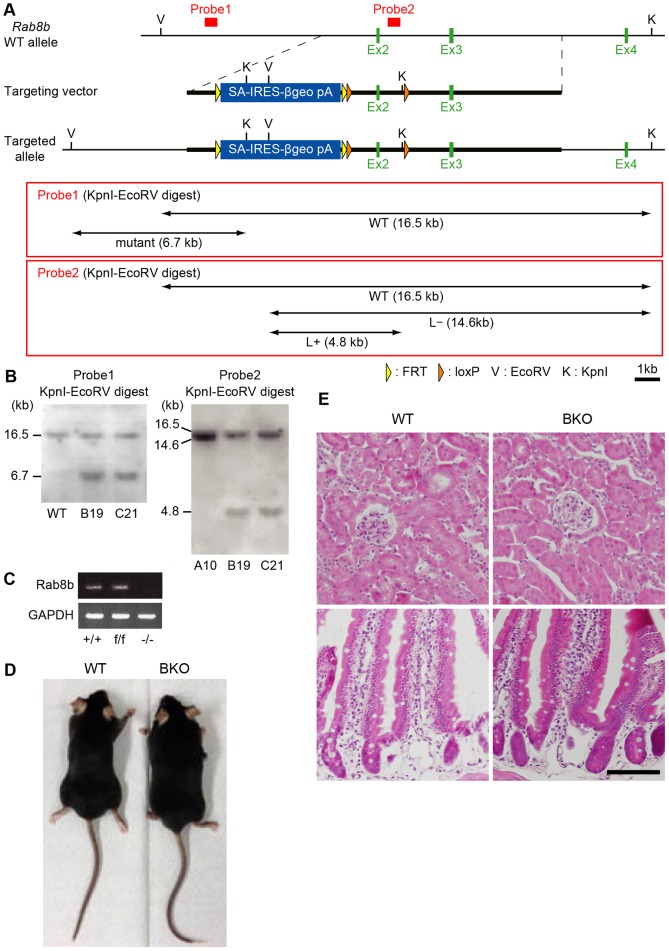
**Generation and analysis of *Rab8b* knockout mice.** (A) Diagram of targeting strategies. Restriction maps of *Rab8b* wild-type (WT) allele, targeting vector with SA-IRES-βgeo-polyA and targeted allele. K, *Kpn*I; V, EcoRV; orange triangle, loxP site; yellow triangle, FRT site. The locations of the 5′ external probe (probe 1) and the 3′ internal probe (probe 2) are indicated. Probe 1 hybridises with a 16.5-kb *Kpn*I-EcoRV fragment from the wild-type allele and with a 6.7-kb fragment from the targeted allele. Probe 2 hybridises with a 16.5-kb *Kpn*I-*Eco*RV fragment from the wild-type locus, a 14.6-kb fragment from the locus that lacks 3′ loxP, and a 4.8-kb fragment from the locus which retains 3′ loxP. (B) Southern blot analysis of the targeted embryonic stem (ES) cell clones. Genomic DNA from the parental ES cells (WT), homologous targeted clones without 3′ loxP (A10), and those with 3′loxP (B19 and C21) were digested with *Kpn*I and EcoRV for hybridisation with probe 1 (left) or for hybridisation with probe 2 (right). (C) RT-PCR analysis of mRNA from *Rab8b^+/+^*, *Rab8bf/f* (Rab8 floxed/floxed mice which only lack SA-IRES-beta geo pA, but two loxP flanking the exon 2 remains) and *Rab8b^−/−^* small intestine. GAPDH was used as an amplification and loading control. (D) Appearance of wild-type (WT) and *Rab8b^−/−^* (BKO) mice. (E) Hematoxylin-Eosin staining of the kidney (top) and small intestine (bottom) from WT and BKO. Scale bar, 100 µm.

**Fig. 2.**
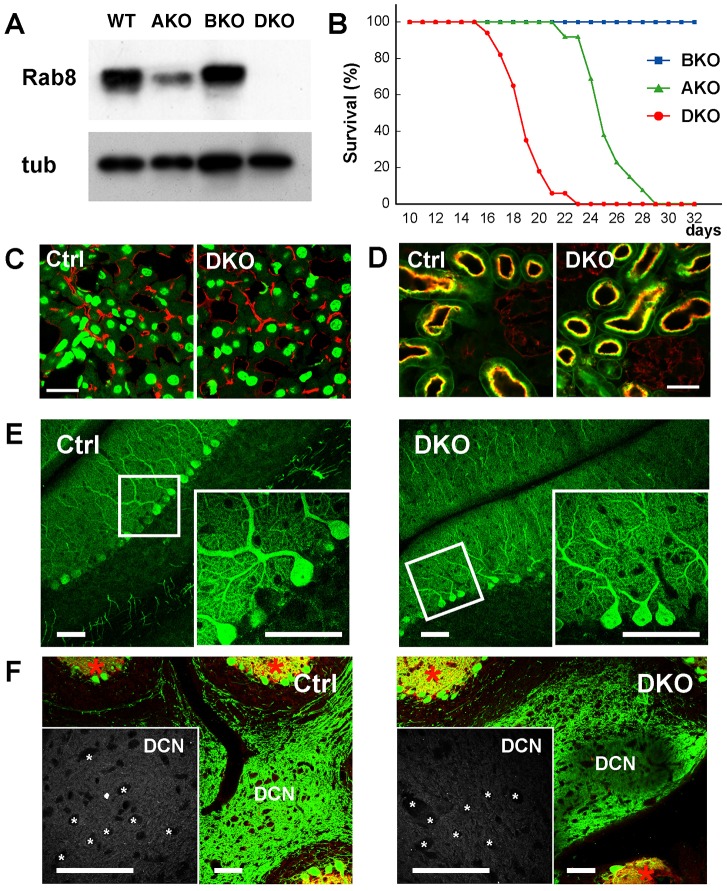
**Phenotype of the *Rab8a* and *Rab8b* double-knockout (DKO) mice.** (A) Western blots of the small intestine lysates from the wild-type (WT), *Rab8a* knockout (AKO), *Rab8b* knockout (BKO), and *Rab8ab* double-knockout (DKO) mice using the anti-Rab8 antibody (Rab8) and an anti-tubulin antibody (tub). (B) Postnatal survival curves of AKO, BKO, DKO mice. DKO mice died ∼1 week earlier than AKO mice. (C) Localisation of the apical marker DPPIV (red) in the liver of the control (Ctrl) and DKO mice at P14. The genotypes of the control mice were *Rab8b*^+/+^, *Rab8b*^+/−^ or *Rab8b*^−/−^ because no phenotypic differences were observed among these genotypes. Nuclei (green) were stained by DAPI. Scale bar: 20 µm. (D) Localisation of the apical marker DPPIV (red) in the kidneys of the control and DKO mice at P14. Proximal tubules (green) were stained by Lotus-lectin. Both markers colocalised at the apical plasma membranes of the proximal tubules. Scale bar: 20 µm. (E) Cerebellar cortex of the control and DKO mice at P14. Soma, axon and dendrites of Purkinje cells were stained with calbindin (green). Inset: Soma and dendrites of Purkinje cells. Scale bars: 50 µm. (F) Deep cerebellar nuclei (DCN) of the control and DKO mice at P14. The dendrite marker GluA1 (red) was not localised at the axonal terminals of the Purkinje cells (green). Inset: neurons (*) in the DCN that receive input from Purkinje cells. Scale bars: 50 µm.

To determine the function of Rab8 in intracellular transport and ciliogenesis, we generated BKO mice (Fig. 1A,B) and confirmed that the *Rab8b* transcript was, indeed, absent in the BKO mice (Fig. 1C). As we were unable to generate a Rab8b-specific antibody, despite a number of trials, we used an antibody that recognised both Rab8a and Rab8b for western blot analysis of the control, AKO, BKO and DKO mice (Fig. 2A). Because we were unable to detect any Rab8 protein in the intestines of the DKO mice, the BKO mice were assumed to be deficient in the Rab8b protein. The BKO mice neither displayed any overt abnormalities (Fig. 1D) nor showed any apparent histological abnormalities in the various tissues analysed (Fig. 1E).

### Rab8a and Rab8b DKO mice do not display any abnormalities in tissues other than the small intestine

To determine whether any redundancy exists between Rab8a and Rab8b, we generated DKO mice. When we intercrossed *Rab8a*^+/−^; *Rab8b*^−/−^ males and females, the DKO mice were born at a Mendelian ratio, but gradually died 2 weeks after birth. Almost all DKO mice had perished by postnatal week 3 (Fig. 2B), approximately 1 week earlier than *Rab8a* single-knockout mice (Sato et al., 2007). To detect abnormalities in the tissues of the DKO mice, we examined their livers and kidneys. Immunofluorescence microscopy of the tissues failed to detect any abnormalities until postnatal week 2 (Fig. 2C,D). Previous results have indicated the involvement of Rab8 in the dendritic transport process in neurons (Huber et al., 1993b). We, therefore, used the cerebellum to test this result *in vivo*. Because Purkinje cells are the only calbindin-positive cells in the cerebellum, the axons of Purkinje cells in the deep cerebellar nuclei (DCN) region can be easily identified. Previously, dentritic markers were found to be mislocalised to the axons of Purkinje cells of mice in which the adaptor protein AP4 had been knocked out (Matsuda et al., 2008). However, we did not observe a mislocalisation of the dendritic marker GluA1 in our DKO mice until postnatal week 2 (Fig. 2F). In addition, the shape of the Purkinje cell dendrites was similar in control and DKO mice (Fig. 2E).

### Several apical markers mislocalise, but basolateral markers localise normally in the small intestine of the DKO mice

**Fig. 3.**
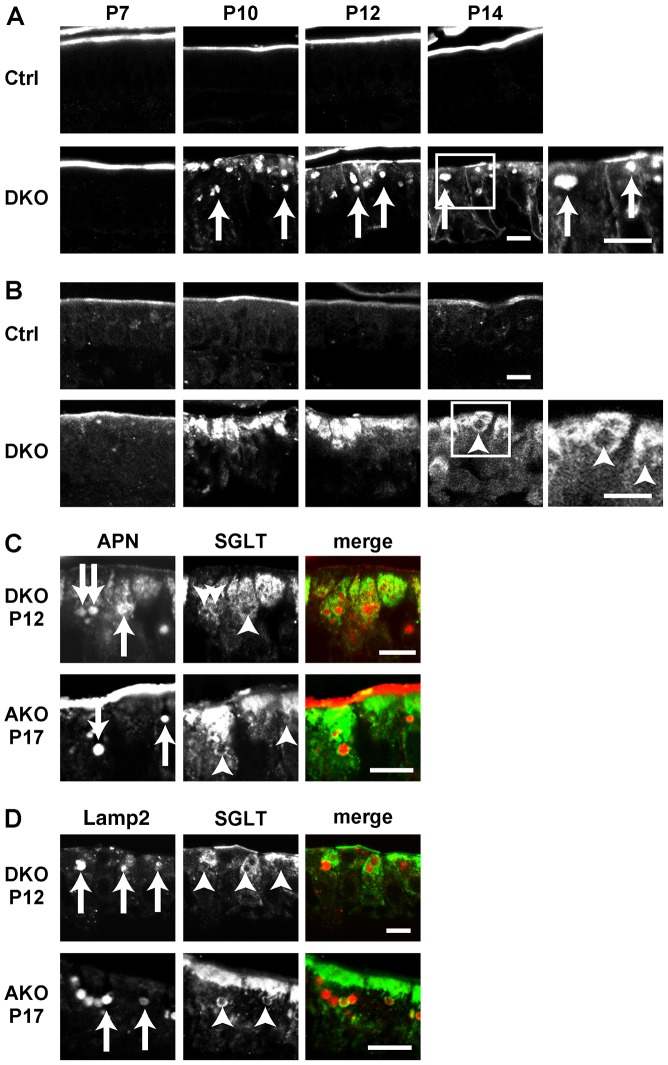
**Localisation of apical markers in the DKO small intestine.** (A,B) Localisation of APN (A) and SGLT (B) in the intestinal epithelial cells from control and DKO mice at postnatal days P7, P10, P12 and P14. High-magnification figures of insets (white boxes) at P14 of DKO are shown to the right of these panels. Intracellular vacuoles of APN (arrows) and SGLT-negative spaces are indicated (arrowheads). (C) Complementary localisation of APN (red) and SGLT (green) in DKO at P12 and AKO at P17. Intracellular vacuoles of APN (arrows) and SGLT-negative spaces are indicated (arrowheads). (D) Complementary localisation of Lamp2 (red) and SGLT (green) in DKO at P12 and AKO at P17. Intracellular vacuoles of Lamp2 (arrows) and SGLT-negative spaces are indicated (arrowheads). Scale bars: 10 µm.

Having previously identified the mislocalisation of apical markers in AKO mice at postnatal day 17 (P17) (Sato et al., 2007), we next examined the small intestine of the DKO mice at different postnatal (P) days. When we examined the localisation of apical markers, such as aminopeptidase N (APN), dipeptidyl peptidase IV (DPPIV) and sodium-dependent glucose transporter (SGLT), their localisation was similar in control and DKO mice small intestine at P7 (Fig. 3A). However, we found intracellular accumulation of these markers in DKO enterocytes from P10, i.e. 1 week earlier than those found in the AKO enterocytes. Interestingly, the pattern of intracellular accumulation differed depending on the apical markers. APN (Fig. 3A, arrows) and DPPIV (supplementary material Figs S1, S2, arrows) displayed punctate accumulation, whereas SGLT displayed a diffuse staining pattern (Fig. 3B). Interestingly, APN and SGLT presented mutually exclusive localisation (Fig. 3C, top). A similar mutually exclusive staining pattern was also observed in the AKO mice at later postnatal ages (Fig. 3C, bottom). Because SGLT was also stained in mutually exclusive manner to Lamp2, SGLT was thought to localise to a structure distinct from the lysosomes (Fig. 3D).

**Fig. 4.**
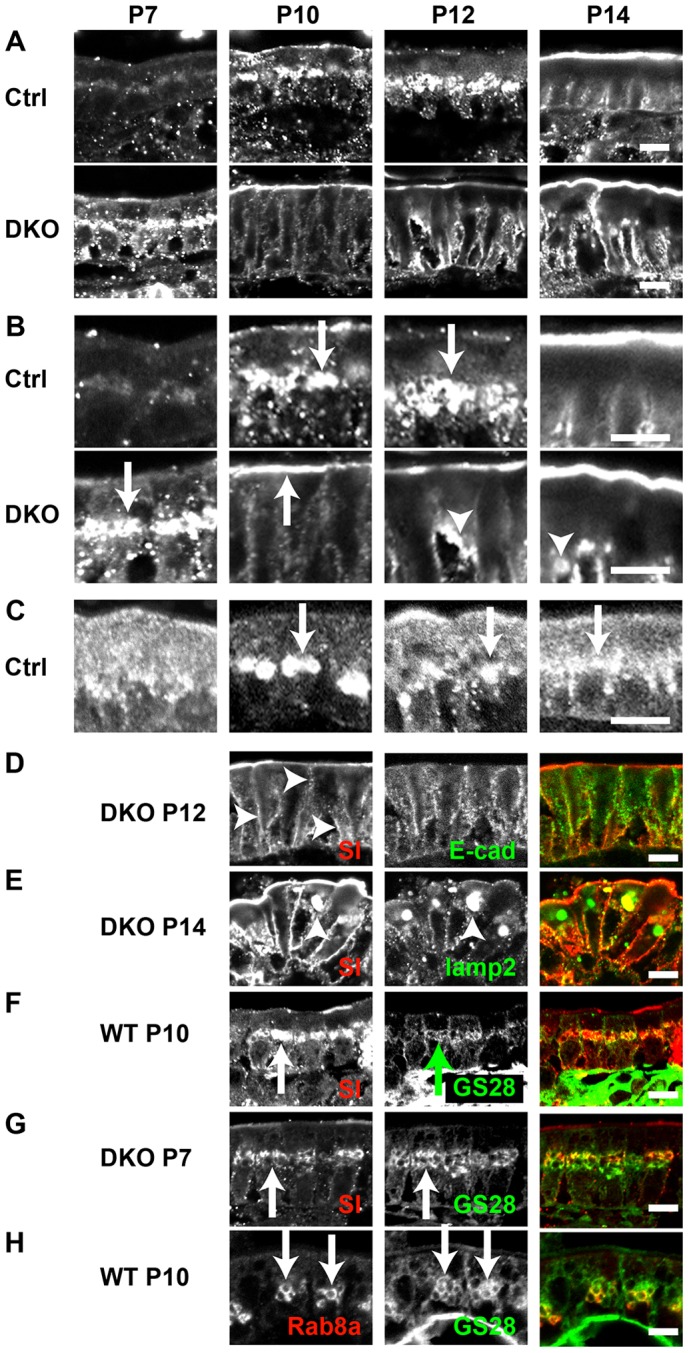
**Localisation of SI and Rab8a in the small intestine.** (A,B) Localisation of SI in the intestinal epithelial cells of the control and DKO mice at different postnatal days at low (A) and high (B) magnifications. Perinuclear staining of SI at postnatal days P7, P10 and P12 is shown by arrows. Intracellular vacuoles in DKO at P14 are shown by arrowheads. (C) Localisation of Rab8a in the control mice at different postnatal days. Perinuclear staining of Rab8a at P10, P12 and P14 are indicated by arrows. (D–G) Comparison of SI (left, red) and other markers (right, green). (D) Double staining of SI and E-cadherin in the DKO intestine at P12. Basolateral staining of SI is shown by arrowheads. (E) Double staining of SI and Lamp2 in DKO intestine at P14. Lysosomal staining of SI is shown by arrowheads. (F) Double staining of SI and Golgi marker, GS28, in WT intestine at P10. Golgi staining of SI is shown by arrows. (G) Double staining of SI and GS28 in the DKO intestine at P7. Golgi staining of SI is shown by arrows. (H) Colocalisation of Rab8a (red) and GS28 (green) in the WT enterocytes at P10 (arrows). Scale bars: 10 µm.

Another apical marker, sucrase-isomaltase (SI), did not – unlike APN or SGLT – accumulate subapically in the DKO mice at P10, but localised to the apical plasma membrane at P14 (Fig. 4A,B). In the DKO, SI began to localise near the nucleus at P7 (Fig. 4B, indicated by an arrow in P7 DKO) and at the apical plasma membrane at P10 (Fig. 4B, indicated by an arrow in P10 DKO). At the later stages, SI mislocalised to the basolateral plasma membrane at P12 (Fig. 4A,B, indicated by arrowheads in P12 DKO and in Fig. 4D) and, finally, to the lysosomes at P14 (Fig. 4B, indicated by an arrowhead in P14 DKO and in Fig. 4E). Perinuclear SI staining at P7 in DKO enterocytes, and at P10 and P12 in control enterocytes (Fig. 4B, arrows in P10 and P12 Ctrl) corresponded to the Golgi complex (Fig. 4F,G; arrows). Interestingly, Rab8a also localised to the Golgi (Fig. 4H, arrows). Colocalisation of Rab8a and SI at these ages suggest that Rab8a interacts with SI at the Golgi to retard the transport of SI. This assumption is supported by the fact that the loss of Rab8 speeds up apical localisation of SI. These observations indicate that there are at least three pathways that lead to the apical plasma membrane.

**Fig. 5.**
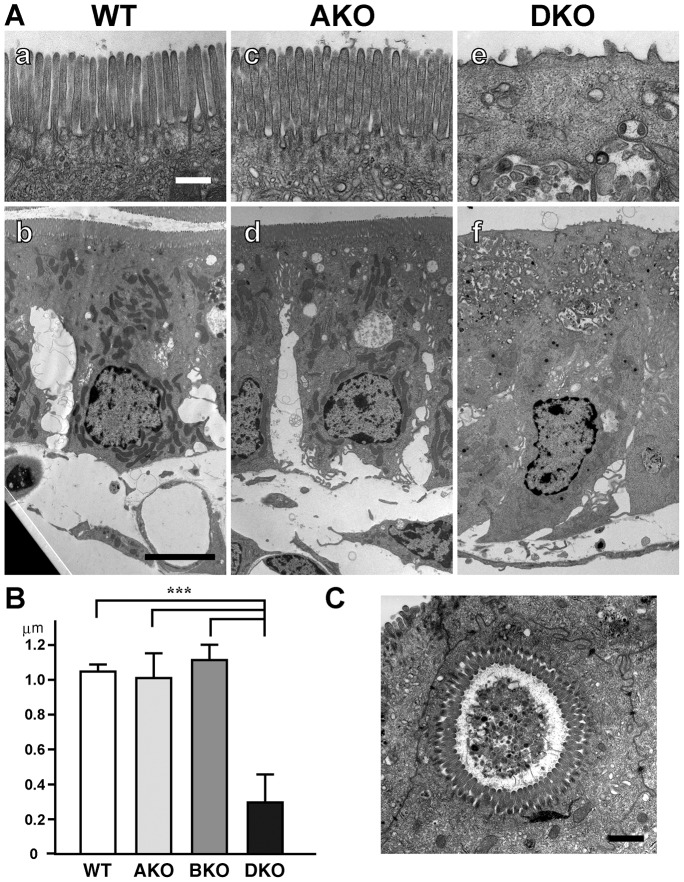
**Microvillus atrophy and inclusions in DKO mice.** (A) Panels a–f show electron micrographs of intestinal cells from postnatal day P14 wild-type (WT) (a,b), *Rab8a* knockout (AKO) (c,d), and double-knockout (DKO) mice (c,f). Apical areas are enlarged (a,c,e). Scale bars: 500 nm (a), 5 µm (b). (B) Graph showing microvillus length of epithelial cells at P14. Values represent the means ± s.d. (*n*>20 cells from three mice per group. ***P<0.001; Student's *t*-test). (C) A microvillus inclusion in a DKO intestinal epithelial cell at P14. Scale bar: 1 µm.

In contrast to apical markers, the basolateral markers did not accumulate intracellularly, as observed in the AKO mice (supplementary material Fig. S2B,C). Thus, both Rab8a and Rab8b are involved in apical transport rather than basolateral transport. The microvillus atrophy-like phenotype of the DKO mice was very similar to that of the AKO mice, although the onset of microvillus atrophy in DKO is about 1 week earlier than the one in AKO mice. When using electron microscopy, we observed a reduction of microvilli (Fig. 5A,B) and the appearance of microvillus inclusion bodies (Fig. 5C) in DKO mice at P14, the time point when AKO mice appeared normal. These phenotypes indicate a synergistic interaction between *Rab8a* and *Rab8b*.

### No abnormalities are found in length and morphology of cilia in DKO mice

Recently, a number of papers have reported that Rab8 is essential for providing ciliary membranes and, thus, for the elongation of cilia (Nachury et al., 2007; Yoshimura et al., 2007; Omori et al., 2008). In recent years it was established that BBS is caused by the mutation of genes, whose products form the BBSome complex, which is essential for ciliogenesis. BBSomes have been shown to interact with Rabin8, a guanine exchange factor for Rab8, indicating the involvement of Rab8 in ciliogenesis (Nachury et al., 2007). Indeed, when Rab8 is knocked down, the lengths of the cilia are reduced (Yoshimura et al., 2007). Thus, to determine the function of Rab8 in ciliogenesis *in vivo*, we observed primary and motile cilia, outer disc membranes and cilium-derived structures in the photoreceptor neurons (Deretic et al., 1995; Moritz et al., 2001).

**Fig. 6.**
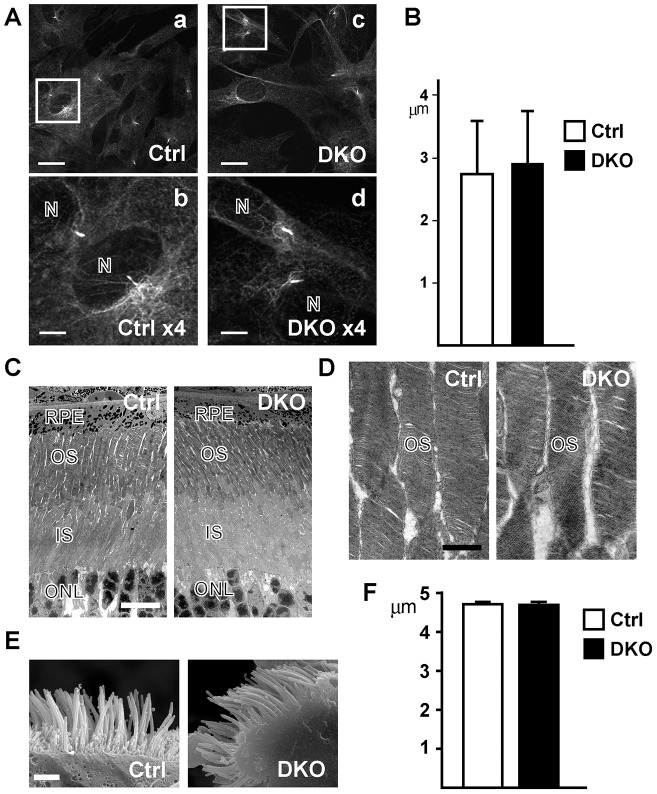
**Primary and motile cilia in control and DKO cells and tissues.** (A) Primary cilia of MEFs stained by acetylated tubulin. (a,b) control, (c,d) DKO. White boxes in top panels (a,c) are enlarged in the bottom panels (b,d). Nuclei (N). Scale bars: 20 µm (a,c), 5 µm (b,d). (B) Graph showing the length of cilia in the MEFs. Values represent the means ± s.d. (*n*>250 cells from three mice per group. *P*>0.02; Student's *t*-test). (C) Electron micrographs of the retina at lower magnifications to show the similar architecture of the control (Ctrl) retina and the DKO retina. (D) Higher-magnification pictures of the retina to highlight the discs in the outer segments. RPE; retina pigment epithelium, OS; outer segment, IS; inner segment, ONL; outer nuclear layer. Scale bars: 10 µm (C), 1 µm (D). (E) Scanning electron micrographs of motile cilia in airway epithelial cells in the trachea. Scale bar: 2 µm. (F) Graph showing lengths of the cilia in the epithelial cells. Values represent the means ± s.d. (*n*>9 cells from three mice per group. *P*>0.02; Student's *t*-test).

**Fig. 7.**
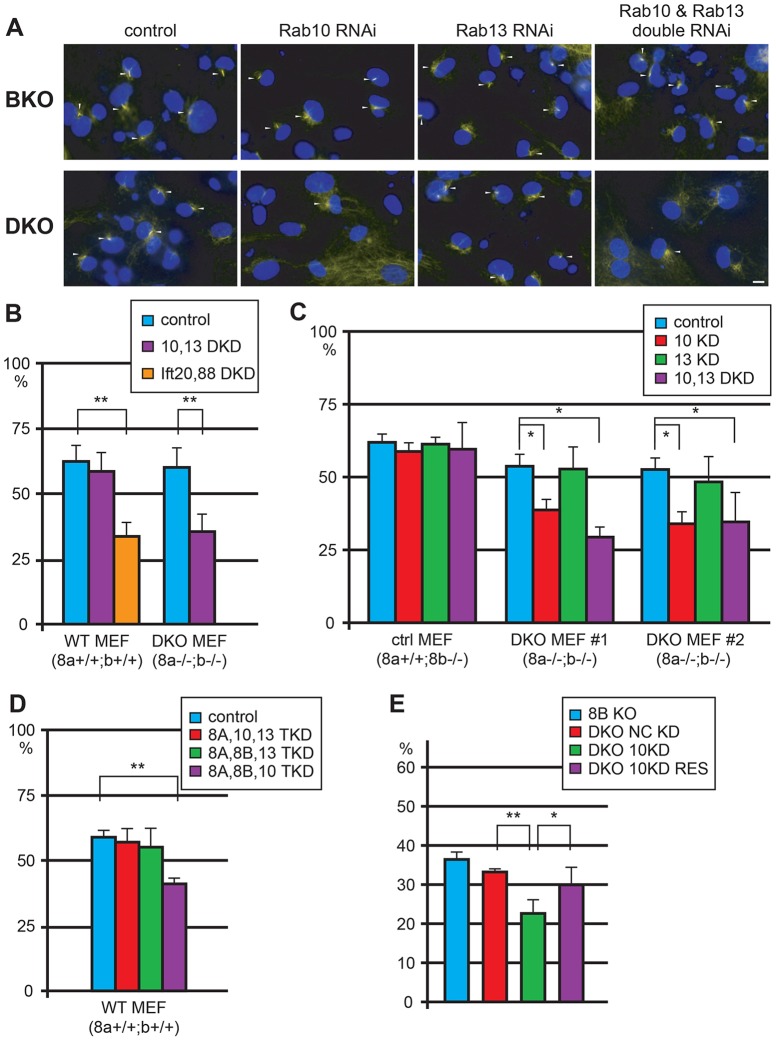
**Rab8a, Rab8b and Rab10 are simultaneously involved in ciliogenesis.** (A) Primary cilia of MEFs whose expression of Rab10, Rab13, and Rab10 and Rab13 together are knocked down by siRNAs are stained with acetylated tubulin (upper) control (BKO), (lower) DKO. Nuclei are stained by DAPI (blue). Cilia are shown by arrowheads. Scale bar: 20 µm. (B) Double knockdown of Rab10 and Rab13 in wild-type and DKO MEFs. Double-knockdown of *Ift20* and *Ift88* ('Ift20, 88 DKD, respectively) effectively decrease the rate of ciliated cells and presented here as a positive control (*n*>150 cells for each genotype. **P<0.01; Student's *t*-test). (C) Knockdown of Rab10 or/and Rab13 in BKO (control) and DKO MEFs (*n*>150 cells for each genotype. *P<0.02; Student's *t*-test). (D) Triple knockdown using different combinations of knockdown oligonucleotides targeting Rab8a, Rab8b, Rab10, and Rab13 in wild-type MEFs (*n*>150 cells for each genotype. **P<0.01; Student's *t*-test). (E) Rescue of the ciliary phenotype by using knockdown-resistant Rab10 cDNA (‘DKO 10KD RES) (*n*>100 cells for each genotype. *P<0.02, **P<0.01; Student's *t*-test).

First, the morphology of the cells and the lengths of the cilia were not significantly different in DKO and control mouse embryonic fibroblasts (MEFs) (Fig. 6A,B). In addition, the percentage of ciliated cells is similar in DKO and control MEFs (Fig. 7A,B). Second, the structures of the retina and the outer segments of the photoreceptors are similar in DKO and control MEFs (Fig. 6C,D). Third, when we examined the olfactory epithelium, we observed a layer of cilia clearly stained by acetylated tubulin (supplementary material Fig. S3), which is in contrast to the observed marked reduction of cilial staining in BBS1- or BBS4-deficient mice (Kulaga et al., 2004). As a representative of a motile cilium, we examined the ciliated epithelium in the trachea using electron microscopy. The overall structures of the ciliated cells were similar in control and DKO MEFs (supplementary material Fig. S3). The ‘9+2’ microtubule structure in the motile cilia was observed in the DKO as well as the control mice (supplementary material Fig. S3, inset). The structure and length of the motile cilia appeared similar in DKO and the control mice (Fig. 6E,F).

From these observations, and the absence of cysts in the kidney (Fig. 1E) and retinal degeneration (Fig. 6A,B), both of which are hallmarks of some ciliopathies, we concluded that Rab8a and Rab8b are not sufficient for the formation of cilia, at least during the lifespan of the DKO mice.

### Rab8a, Rab8b, and Rab10 are all essential for ciliogenesis

Because there are several species of Rabs (Rab10 and Rab13) in the Rab8 family, we knocked down Rab10 and Rab13 together in *Rab8* DKO and control MEFs to determine the functional redundancy within the Rab8 family (supplementary material Fig. S4). When we knocked down these Rabs in DKO MEFs, the percentage of MEFs with primary cilia was greatly reduced (Fig. 7A,B). The percentage was almost equal to that of ciliated MEFs deficient in intraflagellar transport 20 and 88 (*Ift20* and *Ift88* respectively) by double knockdown (Fig. 7B), which is the most potent knockdown treatment on ciliogenesis (Clement et al., 2009). To understand which Rab is more important, we knocked down either Rab10 or Rab13 in Rab8 DKO MEFs (Fig. 7C). We found that Rab10 knockdown is as effective as a double knockdown of Rab10 and Rab13 in DKO MEFs, but Rab13 knockdown had only a subtle effect on ciliogenesis (Fig. 7C). To confirm which combination of Rab8 family proteins does affect ciliogenesis, we used three knockdown oligonucleotides in wild-type MEFs and measured the rate of ciliated cells. Knocking down Rab8a, Rab8b and Rab10 in wild-type MEFs had the most effect on ciliogenesis, whereas other combinations of knocked out Rab proteins had almost no effect (Fig. 7D). Expressing knockdown-resistant Rab10 cDNA into the Rab10 knockdown DKO MEFs rescued the ciliary phenotype (Fig. 7E). Collectively, these results suggested that Rab8a, Rab8b and Rab10 work synergistically for ciliogenesis, whereas Rab13 is not required.

## DISCUSSION

Rab8 has been thought to be involved in basolateral transport in epithelial cells and dendritic transport in neurons (Huber et al., 1993a; Huber et al., 1993b). In addition, several groups have reported that Rab8 is involved in ciliogenesis (Nachury et al., 2007; Yoshimura et al., 2007). Previously, we have shown that Rab8a is essential for apical transport (Sato et al., 2007). However, Rab8a has a closely related isoform, Rab8b. Thus, it is possible that Rab8b functions in basolateral transport. Moreover, the function of Rab8a and Rab8b in neurons and ciliated epithelium was not examined in our previous study. To clarify their roles, we generated Rab8a–Rab8b double-knockout mice and found that both proteins function synergistically in apical transport but do not have a function in basolateral and dendritic transport.

Knocking out both the Rab8 isoforms showed that there are at least three pathways to the apical plasma membrane. The first pathway transports DPPIV and APN. In the absence of Rab8, these cargos accumulate in the lysosomes. The second pathway transports SGLT and in the absence of Rab8, SGLT accumulates in an unknown structure that is different from the lysosomes. The third pathway transports SI. In the DKO intestine, SI is transported to the apical plasma membrane earlier than it is in the control intestine (Fig. 4A,B). As the localisation of SI resembles that of Rab8a in the Golgi, Rab8a might interact with SI in the Golgi, thereby regulating its transport.

Previously, many researchers suggested that Rab8 is essential for ciliogenesis. However, in our current study, when observing *Rab8a* and *Rab8b* DKO mice, the absence of both Rab8 isoforms had no effect on the generation of primary cilia (olfactory epithelium, retina outer segment and MEF) or motile cilia (trachea). As the DKO mice died within 3 weeks after birth, Rab8a and Rab8b might function after the establishment of cilia. Previously, either Rab8 isoform was shown to function by itself in ciliogenesis (Nachury et al., 2007; Yoshimura et al., 2007). However, our results indicate that Rab8a, Rab8b and Rab10 function simultaneously in ciliogenesis rather than alone, whereas Rab13 does not seem to be necessary for the process. The fact that Rab10 is involved in generating cilia in the apical membrane is surprising because it was thought to be involved in basolateral transport (Chen et al., 2006; Schuck et al., 2007). A previous report describes the localisation of Rab10 on the cilia and the interaction of Rab10 with the exocyst complex, but did not present evidence that Rab10 is involved in ciliogenesis (Babbey et al., 2010). The involvement of Rab10 in ciliogenesis in the MEFs might be explained by the fact that MEFs are not polarised. Several papers have described the specialised membranous area that segregates the cilia from the surrounding apical membrane (Nachury et al., 2010). Thus, it is also possible that transport to the basolateral plasma membrane and ciliary membrane use common molecular machinery. For further elucidation of the mechanism of apical transport and ciliogenesis, more studies – including Rab10-knockout studies – are necessary.

Rab10 is also reported to be necessary for axonal membrane trafficking and axonal elongation (Wang et al., 2011b; Liu et al., 2013). Rab13, a Rab closely related to Rab8 and Rab10, is reported to be necessary for neurite elongation (Sakane et al., 2010). Additionally, Rab17 and Rab22, which belong to different subfamilies of Rab8, have been reported to be necessary for dendrite (Mori et al., 2012) and neurite elongation (Wang et al., 2011a), respectively. Thus, no obvious abnormality in the nervous system in Rab8a and Rab8b DKO mice could be explained by compensation through Rab17 and Rab22. Recently, Rab10 has been shown to be involved in various kinds of intracellular trafficking events. Beside any aforementioned role in neurite elongation and ciliogenesis, Rab10 has recently proposed to be involved in ER dynamics (English and Voeltz, 2013) and the fusion of the Glut4 storage compartment to the plasma membrane (Chen et al., 2012). To assess these roles of Rab10 and the fact that Rab10 might compensate the lack of other Rabs *in vivo*, we believe that Rab10-knockout studies in any model organism (e.g. mouse, worm, fly) are necessary, as well as studies that use organisms in which Rab10 and other Rab family proteins are knocked out simultaneously.

## MATERIALS AND METHODS

### Generation of *Rab8b*-knockout mice

All animal procedures were performed within the guidelines of the Animal Care and Experimentation Committee of Gunma and Osaka University, and all animals were bred at the Institute of Animal Experience Research of Gunma and Osaka University.

Generation of *Rab8b* knockout (BKO) mice was performed largely according to a previous report (Harada et al., 1994). *Rab8b* genomic clones were isolated from a mouse genomic BAC library from the 129Sv/J mouse strain (RPCI-22: Children's Hospital Oakland Research Institute, Oakland, CA), using a fragment of the mouse *Rab8b* second intron (indicated as probe 2 in Fig. 1A) as a probe. The targeting vector consisted of a 1-kb 5′ homologous region, a SA-IRES-βgeo-polyA cassette flanked with two FRT sites and a downstream loxP site (Sato et al., 2007), a 1-kb genomic sequence region including exon 2 (from 350 bp upstream of exon 2 to 400 bp downstream of exon 2), a single loxP site, and a 6.7-kb 3′ homologous region. Two targeted clones (B19, C21) were identified by Southern blot analysis using probes 1 and 2 (Fig. 1B,C). To generate the null mice, we crossed *Rab8b* βgeo/+ mice with Act-Flp-e transgenic mice and then with CMV-cre transgenic mice (Jackson Laboratory, Bar Harbor, ME).

### Immunofluorescence

Mice at various postnatal days were anesthetised, injected intracardially with 3% paraformaldehyde (PFA) in 0.1 M phosphate buffer (pH 7.2) and kept for another 2 hours in that same fixative. Fixed tissues were soaked in 4, 10, 15 and 20% sucrose in 0.1 M phosphate buffer (pH 7.2) at 4°C for more than 30 minutes each step for cryoprotection. The tissues were then frozen in isopentane chilled in liquid nitrogen and stored in liquid nitrogen until cryosectioning. The tissues were cut at 5–10 µm, except for the cerebellum (shown in the next section).

Antibodies, dye and lectin used: APN (1∶100; BMA Biomedicals, Augst, Switzerland); DPPIV (1∶100; R&D Systems, Minneapolis, MN); Lotus-lectin (1∶100; Honen, Tokyo, Japan); DAPI (1∶1000; 4′,6-Diamidine-2′-phenylindole dihydrochloride; Roche, Basel, Switzerland); Rab8 (1∶1000 for WB; BD, San Jose, CA); SI (1∶100; a gift from S. Matsumoto; Umesaki et al., 1982); Rab8a (1∶100; Sato et al., 2007); SGLT (1∶100; Santa Cruz, Dallas, TX); Lamp2 (1∶100; clone Abl-93; Developmental Studies Hybridoma Bank, IA); E-cadherin (1∶50; TAKARA, Otsu, Shiga, Japan); and Na^+^-K^+^ ATPase (1∶100; a gift from H. Homareda; Homareda et al., 1993); acetylated tubulin (1∶100; Sigma). We used our antibody for Rab8a for immunofluorescence (Sato et al., 2007) because an antibody that recognises both Rab8a and Rab8b (BD, San Jose, CA) did not work for immunofluorescence in our hands. For secondary antibodies, Alexa-Fluor-488- or Alexa-Fluor-546-labeled species-specific secondary antibodies (1∶400; Life Technologies, Carlsbad, CA) were used.

### Cerebellum staining

Histological assays for mouse cerebellar slices were performed as previously described (Hirai et al., 2005). Briefly, micro-slicer sections were incubated overnight with the following primary antibodies: mouse anti-calbindin (1∶1000; Swant, Marly, Switzerland) and rabbit anti-GluA1 (1∶200; Millipore, Billerica, MA). Sections were incubated for 1 hour with Alexa-Fluor-488- or Alexa-Fluor-546-labeled species-specific secondary antibodies (1∶1000; Life Technologies, Carlsbad, CA). The stained slices were analysed by using confocal microscopy (Olympus, Tokyo, JAPAN) as described previously (Hirai et al., 2005).

### Olfactory epithelium staining

For immunofluorescence microscopy, DKO and control mice were anaesthetised and perfused with phosphate-buffered saline (PBS), followed by 4% PFA in PBS. The nose, containing the olfactory epithelium, was removed, post-fixed for 3 hours in the same fixative, de-calcified in 0.5 M EDTA for 2 days, and cryoprotected by immersion in 20% sucrose (Koshimoto et al., 1992). The frontal sections (20 µm thickness) of the nasal epithelium were obtained using a cryostat and mounted on MAS-coated slide glass (Matsunami, Osaka, Japan). The sections were treated with 0.2% Triton X-100 in PBS containing 5% normal horse serum for 60 minutes and then incubated overnight at room temperature with a rabbit anti-golgin 97 antibody (1∶500; a gift from N. Nakamura; Yoshimura et al., 2004) and mouse anti-acetylated tubulin antibody (1∶1000; Sigma). After washing in PBS, the sections were incubated with 3.75 µg/ml Cy3-conjugated donkey anti-rabbit IgG (Jackson ImmunoResearch, West Grove, PA) and 5 µg/ml Alexa-Fluor-488-conjugated donkey anti-mouse IgG (Invitrogen, Carlsbad, CA) for 60 minutes at room temperature and washed in PBS. The mounted specimens were then examined using confocal laser scanning microscopy (Olympus FV1000, Olympus, Tokyo, Japan) or fluorescence microscopy (Zeiss Axio Observer Z1, Carl Zeiss Japan, Tokyo, Japan).

### Histology and western blot analyses

Eight- to nine-week-old mice were used for histology and immunofluorescence microscopy. Samples were fixed using perfusion with 4% (w/v) PFA in 0.1 M phosphate buffer (pH 7.4) and processed as previously described (Harada et al., 1990, Sato et al., 2011). We performed hematoxylin-eosin staining using standard histological procedures. Western blot analyses were performed as previously described (Sato et al., 2011) and 10 µg of protein was loaded per lane.

### Electron microscopy

Mice were perfused with 2% PFA and 2.5% glutaraldehyde in 0.1 M cacodylate buffer (pH 7.4). Tissues were dissected and further fixed for 2 hours at RT and treated with 1% OsO4 in 0.1 M cacodylate buffer followed by 0.5% uranyl acetate in water. The samples were dehydrated and embedded in Epon, and thin sections were post-stained with uranyl acetate and lead citrate as described previously (Harada et al., 1990). Sections were then studied under an electron microscope (model 1010; JEOL, Tokyo, Japan) at 80 kV.

For scanning EM, fresh tissues were dissected out and fixed for 4 hours in 2% PFA and 2.5% glutaraldehyde in 0.1 M cacodylate buffer (pH 7.4). Tissues were further fixed in 1% tannic acid in 0.1 M cacodylate buffer for 2 hours and 1% OsO_4_ in 0.1 M cacodylate buffer for 1 hours. Tissues were then transferred to 50% DMSO in water, frozen and fractured in liquid nitrogen. The samples were substituted in t-butyl alcohol, freeze-dried and sputter-coated with Pt-Pd (E-1010; Hitachi High-Technologies Corporation, Tokyo, Japan). Samples were viewed at 15 kV using scanning electron microscopy (S-4100; Hitachi High-Technologies Corporation).

### Cell culture

Control and DKO mice, generated by intercrossing *Rab8a*^+/−^; *Rab8b*^−/−^ mice, were dissected on embryonic day 13.5, and MEFs isolated by trypsinisation of the embryos as described previously (Uemura et al., 2009). MEFs were cultured in Dulbecco's modified minimal Eagle's medium (DMEM) supplemented with 10% foetal calf serum (FCS) at 37°C in a humidified 5% CO_2_ 95% air atmosphere. To stain cilia, MEFs were fixed in 3% PFA in PBS for 15 minutes at RT, permeabilised in 0.05% saponin and then stained with an anti-acetylated tubulin antibody.

### Knockdown and measurement of cilia

To analyze cilia, 2.5×10^5^ MEFs were cultured on a 12-mm round coverslip in normal culture medium for 2 days and in DMEM containing 0.5% FCS for an additional 2 days. After starvation, cells were fixed with 4% PFA in PBS for 20 minutes at RT. Cells were treated with methanol∶acetone (1∶1) solution at −20°C for 15 minutes, followed by permeabilisation with 0.5% Triton X-100 in PBS. The permeabilised cells were incubated with the blocking solution (5% normal donkey serum and 0.01% Triton X-100 in PBS) for 1 hour at RT, then with the blocking solution containing mouse anti-acetylated tubulin monoclonal antibody (1∶4000; T6793, Sigma), overnight at 4°C. Cells were washed with 0.01% Trion X-100 in PBS and incubated with Alexa-Fluor-488-conjugated anti-mouse IgG (1∶200; Invitrogen, Carlsbad, CA) and DAPI (1∶1000) for 45 minutes. After washing with PBS, the coverslips were mounted and viewed on a microscope. For knockdown experiments, 100 pmol small interfering RNAs (siRNAs) targeting *Rab8a*, *Rab8b*, *Rab10*, *Rab13*, *Ift20* and *Ift88* (Thermo Scientific Dharmacon, Lafayette, CO, siGENOME set of four; catalogue numbers MQ-040860-00-0002, MQ-055301-00-0002, MQ-040862-01-0002, MQ-045749-01-0002, MQ-050410-01-0002 and MQ-050417-00-0002) was electroporated into 5×10^5^ MEF cells. One 100 V pulse of 10 mseconds and subsequent five square driving pulses (20 V) of 50 mseconds, at 50-msecond intervals, were applied using a pulse generator CUY21 edit II and a cuvette (SE-202P, BEX, Tokyo, Japan). In the rescue experiment for Rab10 knockdown, 8 µg of FLAG-tagged human Rab10 subcloned into pcDNA5 plasmid (Life Technologies) and 2 µg of pEGFP-N2 plasmid (Clontech) were electroporated into MEF cells together with the relevant siRNA. In the human Rab10 gene, we designed silent mutations for the resistance against mouse Rab10 siRNAs. The percentage of ciliated cells was calculated in electroporated cells that express EGFP.

### Image processing and quantifications

Images were processed using Adobe Photoshop® (Adobe Systems, Inc., San Jose, CA) version 7.0.

## Supplementary Material

Supplementary Material
