# Supplementary material for: Rab8a and Rab8b are essential for several apical transport pathways but insufficient for ciliogenesis
Source: J Cell Sci. 2014 Jan 15;127(2):422–31. doi: 10.1242/jcs.136903 (PMC3898603; doi:10.1242/jcs.136903)
Supplement: Supplementary Material [file supp_127_2_422__index.html]

Supplementary Material 

# Rab8a and Rab8b are essential for several apical transport pathways but insufficient for ciliogenesis

## JCS136903 Supplementary Material

**Files in this Data Supplement:**

- **Supplementary Material PDF**
