# Supplementary material for: Rab8a and Rab8b are essential for several apical transport pathways but insufficient for ciliogenesis
Source: J Cell Sci. 2014 Jan 15;127(2):422–31. doi: 10.1242/jcs.136903 (PMC3898603; doi:10.1242/jcs.136903)
Supplement: Supplementary Material [file supp_jcs.136903_JCS136903.pdf]

Supplementary figure 1

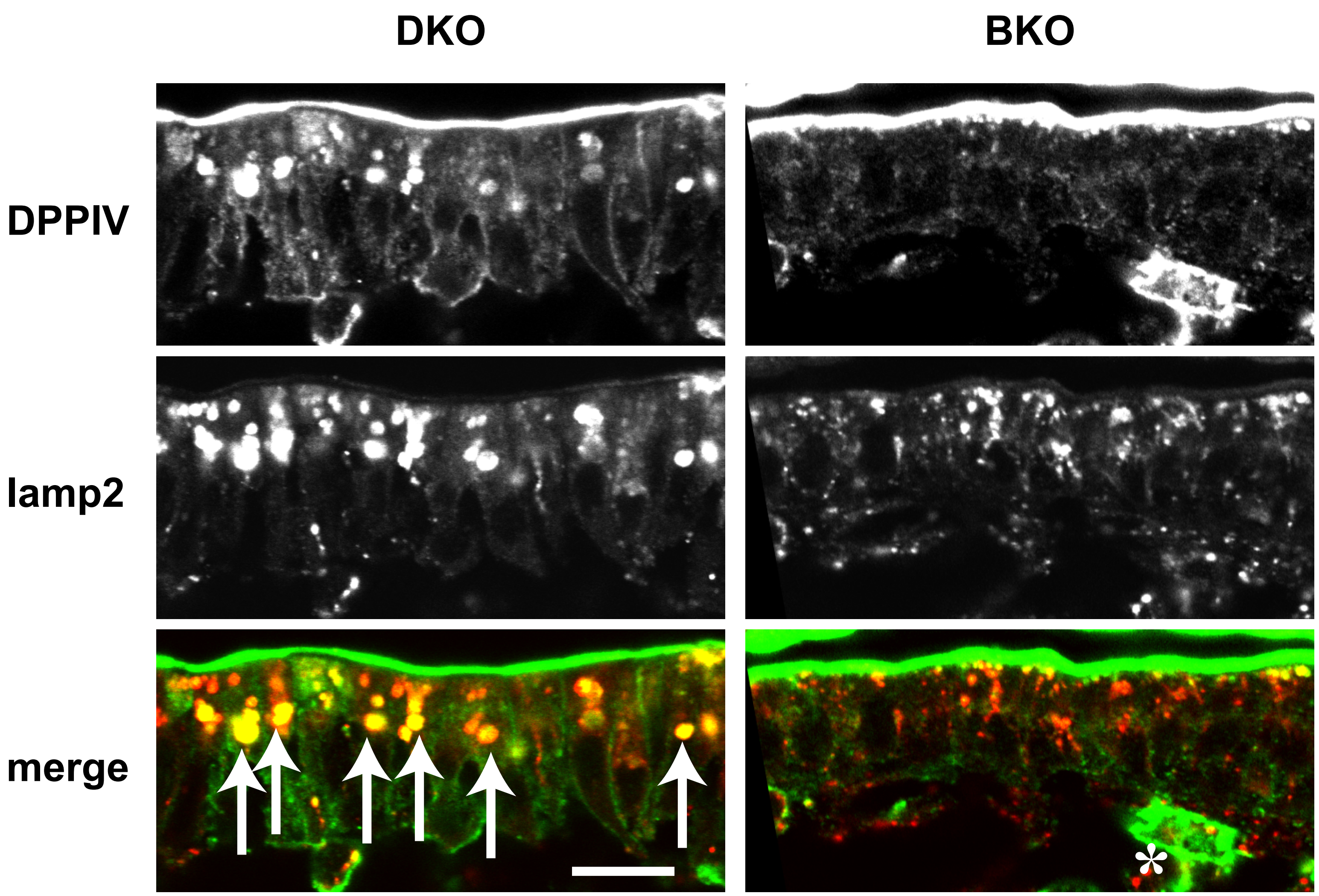

Fig. S1. **Colocalization of an apical marker (DPPIV) and a lysosomal marker (lamp2) in the epithelial cells in the small intestine from P12 Rab8ab double knockout (DKO) mice.**
Large intracellular vacuoles of DPPIV colocalized with lamp2 are observed only in a villi of DKO intestine (arrows). Mesenchyme underneath the epithelial cells is stained in BKO (asterisk).
Bar, 20µm.

Supplementary figure 2

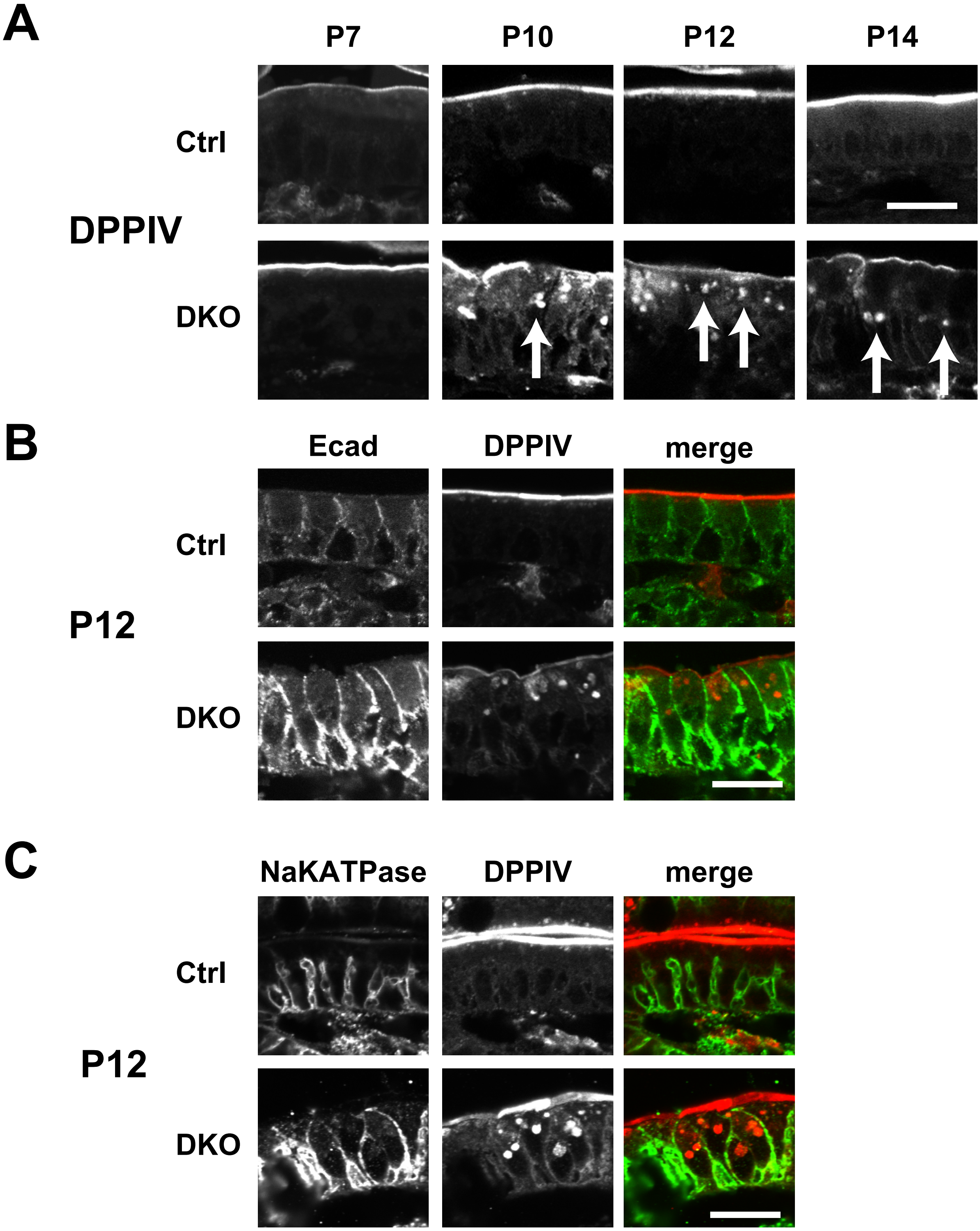

Fig. S2. **Localization of an apical marker (DPPIV) and basolateral markers (E-cadherin and Na K ATPase) in the epithelial cells in the small intestine from control (Ctrl) and DKO mice.**
(A) Localization of DPPIV in control (Ctrl) and DKO mice at P7-P14. Intracellular vacuoles of DPPIV are shown by arrows.
(B) Localization of E-cadherin (green) and DPPIV (red) at P12.
(C) Localization of Na K ATPase (green) and DPPIV (red) at P12. Bars, 20µm.

Supplementary figure 3

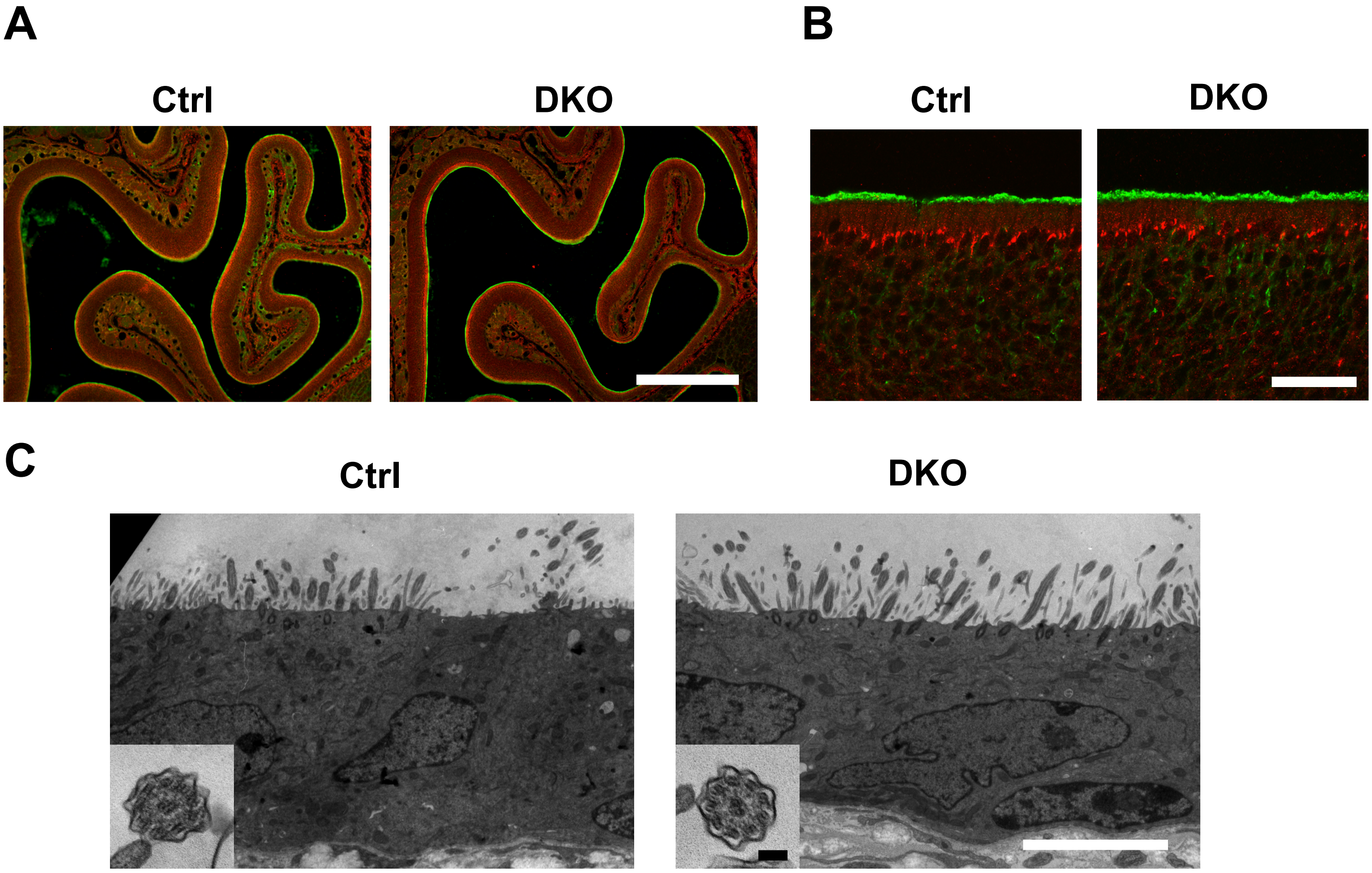

Fig. S3. **Morphology of primary and motile cilia.** (A) Morphology of nasal epithelium in control (Ctrl) and DKO mice at P14. Acetylated tubulin (green), golgin97 (red). Bar, 500μm. (B) High magnification of Figure S3A. Bar, 100μm (C) Electron micrographs of airway epithelial cells from P14 control (Ctrl) and DKO trachea. Cross sections of motile cilia were shown in the inset. Bars, 10μm and 0.2μm (inset).

Supplementary figure 4

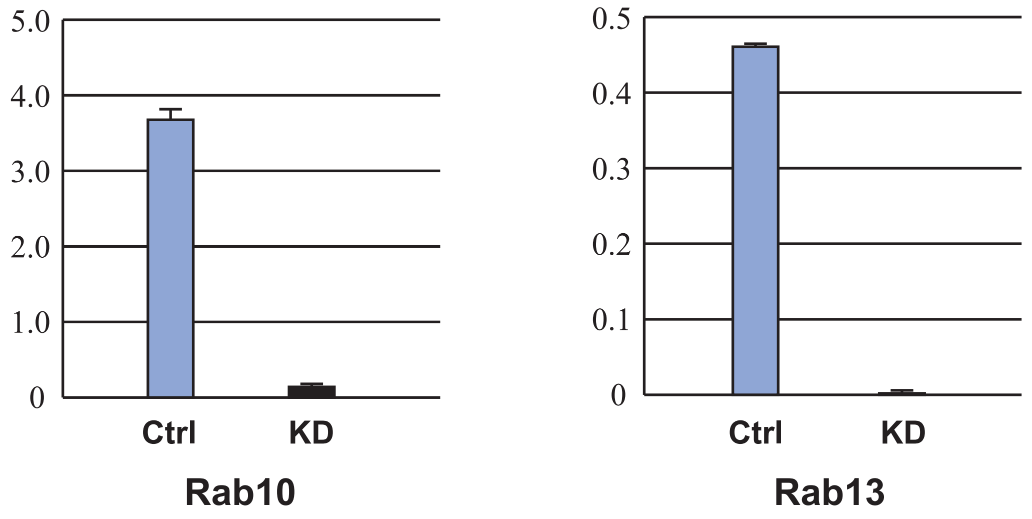

Fig. S4. **Knockdown efficiency measured by real-time PCR.**
The amounts of Rab10 (left) and Rab13 (right) mRNA are measured by real-time PCR. Both of them are greatly reduced after siRNA treatment.
